# Arsenic Trioxide Cooperate Cryptotanshinone Exerts Antitumor Effect by Medicating Macrophage Polarization through Glycolysis

**DOI:** 10.1155/2022/2619781

**Published:** 2022-02-08

**Authors:** Tao Jiang, Jian-Bo Huang, Chu-Yu Xu, Yuan-Lin Lv, Jun Lu, Zheng-Qi Zhao, Dan-Qian Yang, Zhao-Huan Lou, Guang-Ji Zhang

**Affiliations:** ^1^College of Basic Medical Sciences, Zhejiang Chinese Medical University, Hangzhou, China; ^2^Key Laboratory of “Blood Stasis and Toxin” Syndrome of Zhejiang Province, Hangzhou, China; ^3^The First Affiliated Hospital of Zhejiang Chinese Medical University, Hangzhou, China; ^4^College of Medical Technology, Zhejiang Chinese Medical University, Hangzhou, China; ^5^College of Pharmaceutical Sciences, Zhejiang Chinese Medical University, Hangzhou, China

## Abstract

Hepatocellular carcinoma (HCC) is an often-fatal malignant tumor with high lethality. Despite advances and significant efficacy in monotherapy, cancer therapy continues to pose several challenges. Novel combination regimens are an emerging strategy for anti-HCC and have demonstrated to be effective. Here, we propose a potential combination for HCC treatment named arsenic trioxide cooperate cryptotanshinone (ACCS). A remarkable synergistic therapeutic effect has been achieved compared with drugs alone in both in vivo and in vitro experiments. Mechanism study indicated that ACCS exerts its therapeutic actions by regulating macrophage-related immunity and glycolysis. ACCS potentiates the polarization of M1 macrophages and elevates the proportion of M1/M2 to remodel tumor immunity. Further molecular mechanism study revealed that ACCS intensifies the glucose utilization and glycolysis in the macrophage by increasing the phosphorylation of AMPK to activating the AMPK singling pathway. In conclusion, ACCS is a highly potential combination regimen for HCC treatment. The therapeutic potential of ACCS as a candidate option for anticancer drugs in restoring the balance of immunity and metabolism deserves further investigation.

## 1. Introduction

Hepatocellular carcinoma (HCC) is one of the most prevalent malignancies and the fourth leading cause of tumor-associated mortalities worldwide [1]. Intense efforts have been devoted to develop new chemotherapeutic agents in the clinic. Arsenic is a potential candidate for adjuvant therapies [2] of advanced HCC with a long history of being used as a pigment and poison for the human body and tissue. Currently, arsenic trioxide (ATO) was highly recommended for leukemia and serves as the first-line treatment for acute promyelocytic leukemia (APL) [3]. Differing from the promising therapeutic effects in APL, the outcome of HCC patients treated with ATO is unsatisfactory. One of the major reasons is that the rapid clearance of ATO in the blood cannot give a satisfactory accumulation in the solid tumor which means that for ATO to give a considerable effect, the dose almost exceeds the safety and recommended range [4, 5]. Now, the use of combination therapies, where several antitumor agents are used together, is a promising strategy for further clinical use of ATO [6]. Natural products have been found to have immunomodulatory effects in a variety of complex immune-related diseases including tumors and ulcerative colitis [7–9]. We addressed an underlying novel natural plant extract called cryptotanshinone (CTS), which is extracted from *Salvia miltiorrhiza*, is a potential therapeutic agent in cancer and a partner for ATO. In our previous studies, we have confirmed the effects of ATO cooperate CTS (ACCS) in transplanted tumor, but the underlying mechanism is not clear [6, 10].

Tumor-associated macrophages (TAMs) in the microenvironment play critical and complex roles in tumor progression. Macrophages can be polarized into the M1 or M2 phenotype in response to different stimuli. M1 macrophages have remarkable proinflammatory effects and tumoricidal activity [11]. Conversely, M2 macrophages are anti-inflammatory and have protumor activity via promoting tumor cell proliferation, migration, angiogenesis, and the immunosuppressive microenvironment. Changes in the phenotype in TAMs lead to vastly different consequences in tumor progression, and it is a critical issue to reverse the abnormal activation in cancer research.

Metabolism in TAMs can be an entry point for changing macrophage polarization. M1 and M2 display different metabolic phenotypes obviously; M1 relies on glycolysis while M2 relies primarily on oxidative phosphorylation (OXPHOS) [12]. For this reason, boosting glucose uptake and switching the metabolism from OXPHOS to glycolysis are valid actions to reverse the polarization of macrophages. Several studies have shown evidence that ACCS has the ability to alter the immune system by manipulating macrophages [13], and it can also affect the metabolic-related biofunction [14, 15]. Hence, it is interesting to explore whether ACCS could regulate macrophage polarization and tumor immunity function as metabolic regulators in HCC.

## 2. Materials and Methods

### 2.1. Cell Culture

The Hepa1-6 cell line was purchased from the National Collection of Authenticated Cell Cultures of China (NCACC). The H22 cell line was gifted by the Zhejiang Chinese Medical University Laboratory Animal Research Center (ZUMU-LARC). Peritoneal macrophages were extracted from the peritoneal cavity in mice. H22 cells and peritoneal macrophages were cultured with the RPMI 1640 Medium (Gibco, CA, USA) with 10% fetal bovine, 1% penicillin, and streptomycin, grown at 37°C with 5% CO_2_. Hepa1-6 cells were grown in DMEM (Gibco, CA, USA) with 3.5% sodium bicarbonate, 10% fetal bovine, and 1% antibiotic added.

### 2.2. Cell Proliferation Assays

Cell in vitro proliferation was determined by the CCK8 experiment following the manufacturer's instructions. Briefly, cells were seeded in 96-well plates and cultured with ATO (Shuanglu, Beijing, China) and CTS (Yuanye Bio-Technology, Shanghai, China) for 24 hours. For ACCS groups, different drug combinations were set up to search for the best ration of ATO and CTS. Following incubation for 24 hours, the CCK8 solution (BOSTER, Wuhan, China) was added and incubated at 37°C for 1 hour. The absorbance was detected at 570 nm.

### 2.3. Tumor Inhibition Experiment In Vivo

40 BALB/c mice were ordered from the Shanghai SLAC Laboratory Animal Co., Ltd. Mice were bred and maintained in SPF conditions with a 12 h light/12 h dark cycle, clean diet, and plenty of food. All animal experiments were approved by the IACUC of the Zhejiang Chinese Medical University. After a week of acclimatization, mice were inoculated with 5 × 10^5^ tumor cells and randomly assigned to 4 groups: CON (1% carboxymethyl cellulose sodium, oral administration), ATO (2.5 mg/kg, intraperitoneally), CTS (40 mg/kg, oral administration), and ACCS (2.5 mg/kg ATO, intraperitoneally, and 40 mg/kg CTS, oral administration). The doses of ATO and CTS were derived from our previous study [10]. Drug treatment was started when the tumor became palpable. Treatments were continued for 21 days, and tumor volume was recorded.

### 2.4. Polarization of Macrophages in Tumor Tissues

The polarization of macrophages was detected by flow cytometry after the preparation of the single-cell suspension from tumors. F4/80-BV605 (BD Biosciences, CA, USA), CD45-PE-Cy7 (BD Biosciences, CA, USA), and CD11b-AF-700 (BD Biosciences, CA, USA) were used together to identify the mononuclear immune cells in the tumor, then the proportion of CD86-BV421- (BD Biosciences, CA, USA) positive cells was identified as M1, and CD206-FITC- (Invitrogen, Carlsbad, USA) positive cells were identified as M2.

### 2.5. Polarization of Peritoneal Primary Macrophages In Vitro

Primary macrophages were isolated and purified from the peritoneal cavities of mice. In brief, C57BL/6J mice were injected with 2 mL of 3.8% thioglycollate medium (Sigma-Aldrich, MO, USA) intraperitoneally. After 72 hours, mice were euthanized by CO_2_ inhalation, and peritoneal cells were collected by peritoneal lavage with 5 mL precooled PBS. Then, cells were filtered at 300 × *G* for 5 mins and resuspended in 5 mL of serum-free RPMI 1640 medium. Immediately after, cells were plated into 35 mm culture dishes. After 45 mins, cells were washed twice with sterile PBS to remove nonadherent cells. The retained cells were cultured in a complete medium containing antibiotics, and flow cytometry was used to phenotypically characterize by F4/80 and CD113b and positive CD45. For the establishment of the M2 macrophage phenotype, 10 ng/mL IL-4 (PeproTech, NJ, USA) was added into the medium for 24-hour stimulation. For two different cell models, 1 *μ*M ATO, 10 *μ*M CTS, and the cointervention were used for the cell culture. The polarization of macrophages was detected by the procedure previously described.

### 2.6. Glycolysis Measurement in Macrophages

Lactic acids, the main products of glycolysis, were measured using an ELISA (Meimian Biotech, Yancheng, China) kit. The mRNA levels of Glut1 were detected using RT-PCR. The flow of the experiment was performed according to the routine procedure. The key rate limiting enzymes of glycolysis including pyruvate dehydrogenase kinase 1 (PDK1), lactate dehydrogenase A (LDHA), and phosphoglycerate kinase 1 (PGK1) were similarly detected using RT-PCR. The primers are listed in Table 1. Glut1 protein levels were detected using Western Blot with the antibody of Glut1 (1 : 200, Abcam, Cambridge, UK) and analyzed using image for gray-scale values.

### 2.7. Detection of HIF-1*α*/NF-*κ*B and AMPK Signaling Pathway

The activation of HIF-1*α*/NF-*κ*B and the AMPK signaling pathway was determined by the level of related gene expression and protein phosphorylation. The protein levels of p-NF-*κ*B P65 (1 : 1000, CST, MA, USA), NF-*κ*B P65 (1 : 1000, CST, MA, USA), p-I*κ*B*α* (1 : 1000, CST, MA, USA), I*κ*B*α* (1 : 1000, CST, MA, USA), HIF-1*α* (1 : 2000, Abcam, Cambridge, UK), p-AMPK (1 : 1000, CST, MA, USA), and AMPK (1 : 500, Abcam, Cambridge, UK) were detected by Western Blot. The activator of AMPK and AICAR (MCE, NJ, USA) and the inhibitor of AMPK doxorubicin hydrochloride (MCE, NJ, USA) were used to prove the activation of the AMPK signaling pathway.

### 2.8. Statistics

All statistical analyses were conducted by using the SPSS 16.0 software (Chicago, IL, USA) and GraphPad Prism 5.0. Results are expressed as mean ± SD. Student's *t*-test was used to determine the significance of difference between different groups. *p* < 0.05 was considered significant.

## 3. Results

### 3.1. ACCS Inhibits the Progression of HCC In Vitro

**Figure 1:**
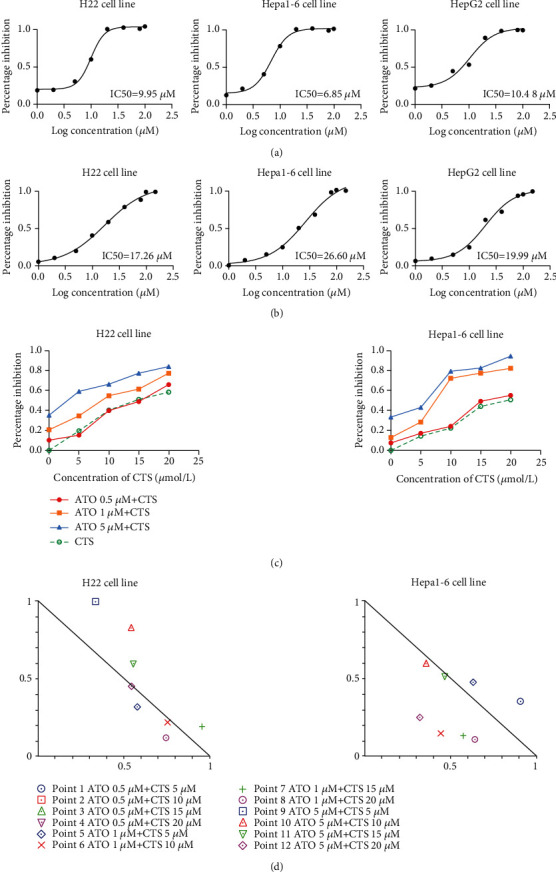
Inhibition curve of different HCC cells. (a) Cells treated with ATO. (b) Cells treated with CTS. (c) Cells treated with the combination of ATO and CTS (ACCS). (d) Normalized isobologram for ACCS.

In order to characterize the effects of ACCS treatments, different HCC cells including those of human origin and mouse origin were used. We first assessed the working effects of monotherapy in ATO and CTS. The results elucidated that both ATO and CTS have an efficiency antitumor effect, and ATO shows a better dose-response relationship. In the H22, Hepa1-6 and HepG-2 cell lines, the IC50 are 9.95 *μ*M, 6.85 *μ*M, and 10.48 for ATO (Figure 1(a)). Similarly, these cell lines are sensitive to CTS, and IC50 values are 17.26 *μ*M (H22), 26.60 *μ*M (Hepa1-6), and 19.99 *μ*M (HepG-2) (Figure 1(b)). Taken together, H22 cells are more sensitive to both drugs and are good models for follow-up studies. Although ATO has a good intervention in tumor growth, the side effects in the solid tumor caused by excess dose limit its future application [16]. Based on this, we chose a lower and dynamic dose to hunt for the possibility of reducing the dosage of ATO. The result illustrated that ATO shows only 10% inhibition at 0.5 *μ*M, and the addition of CTS has no significantly synergistic effects. Inversely, when dose escalation of ATO proceeded up to 1 *μ*M, the cooperation of CTS and ATO illuminated a surprising efficiency in antitumor (Figure 1(c)). We used CompuSyn to calculate the combination index (CI) to measure the synergistic effects of ATO and CTS [17]. Similarly, the results showed that when ATO is at the dosage of 0.5 *μ*M, the combination of the two drugs has no obvious synergistic but antagonistic effects (CI>1). When ATO was enhanced to 1 *μ*M, there was a significant synergistic effect for both 10 *μ*M CTS and 20 *μ*M CTS (Figure 1(d)). This proved that ACCS has a good antitumor effect in vitro.

### 3.2. ACCS Inhibits the Progress of HCC In Vivo

**Figure 2:**
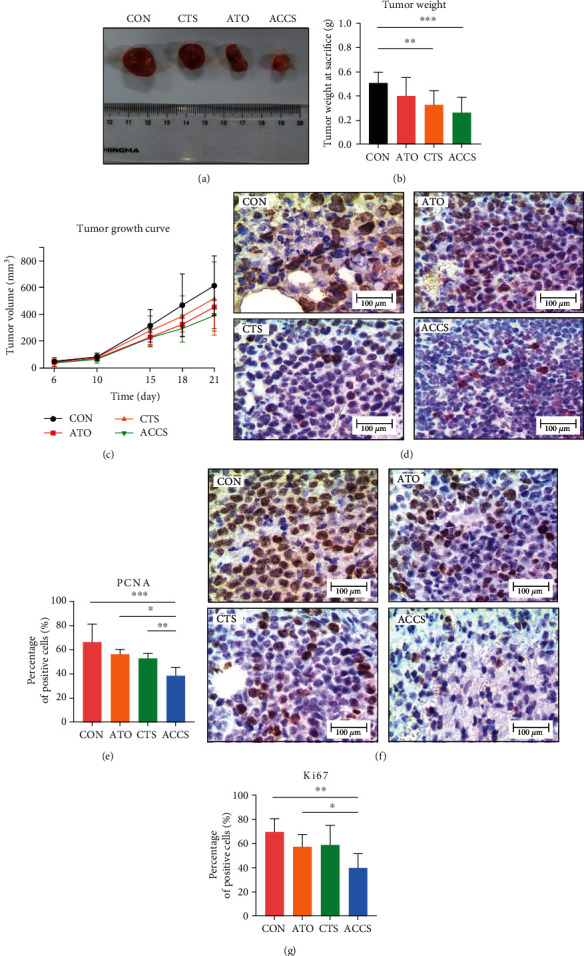
The effects of ACCS in H22 mice xenograft tumors. (a) Representative image of tumor. (b) Tumor weight at 21 days. (c) Tumor growth inhibition curve after treatment. (d, e) Immunohistochemistry of PCNA in tumor tissue. (f, g) Immunohistochemistry of Ki67 in tumor tissue.

The in vitro trial indicated a preliminary efficiency of ACCS in HCC treatment; we further used a xenograft tumor model for validation. Dynamic tumor volume and tumor weight were recorded throughout the experiment. The result demonstrated that ACCS showed a better antitumor effect compared to the single drug usage (Figures 2(a)–2(c)). The expressions of PCNA and Ki67 were also performed to check the proliferation of tumor cells. The results manifested that ACCS can significantly suppress the vitality of tumor cell in vivo by reducing the expression of PCNA and Ki67 (Figures 2(d)–2(g)). These results confirmed that ACCS has an antitumor effect, and the combination is much better than monotherapy. We also detected the routine blood test in mice treated with ACCS. The result showed that no obvious side effects were observed within the treating time (Table 2). Not only that, the use of CTS indicated the potential for ameliorating the side effects in the blood caused by ATO. Notably, we found ACCS significantly increased the counts and ratio of monocytes and neutrophils, implying that ACCS could modulate the immune status of HCC mice. All these results suggested that the cooperation between ATO and CTS has good clinical effectiveness and safety in HCC treatment. Additionally, the therapeutic effect of ACCS is very likely to be related to monocytes and immune regulation.

### 3.3. ACCS Regulates Macrophage Polarization

**Figure 3:**
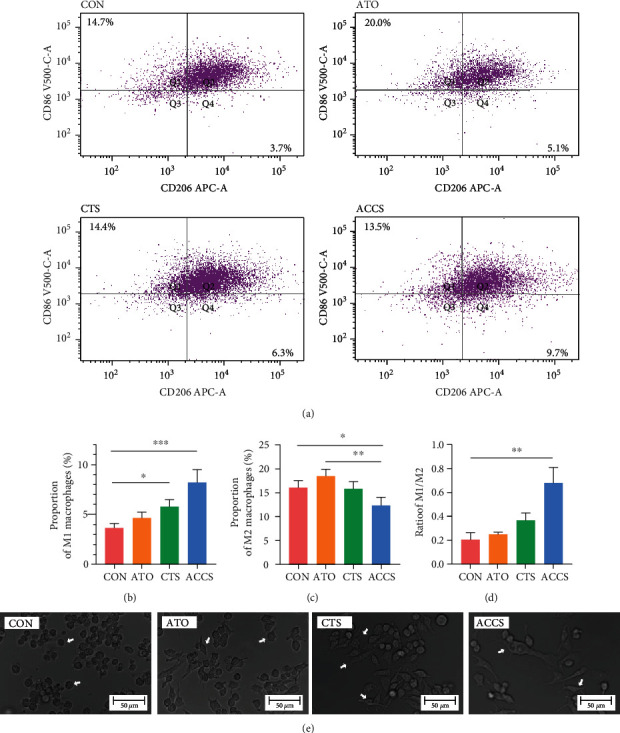
The effect of ACCS on the polarization of macrophages. (a) Macrophages in tumor tissues. (b) The proportion of M1. (c) The proportion of M2. (d) The ratio of M1/M2. (e) Primary macrophages under different drug interventions.

**Figure 4:**
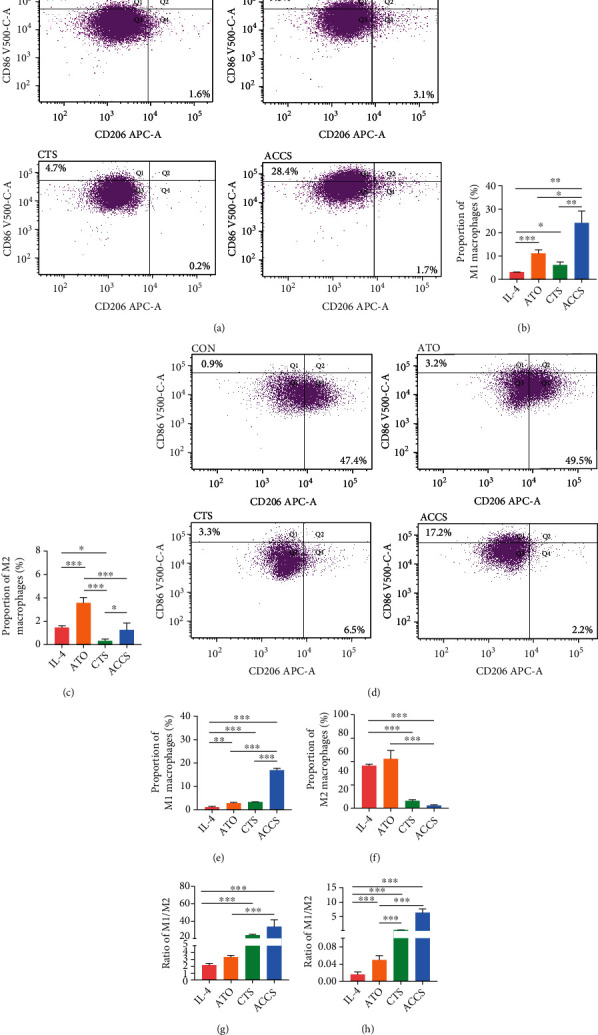
(a) The effect of ACCS on the polarization of primary macrophages. (b) The proportion of M1. (c) The proportion of M2. (d) Tumor-like M2 macrophage model induced by IL-4. (e) The proportion of M1. (f) The proportion of M2. (g) The ratio of M1/M2 in normal group. (h) The ratio of M1/M2 in IL-4-induced group.

In the above study, we found significant changes in monocytes after drug administration. As a predecessor of macrophages, the changes in monocytes imply that ACCS may have a potential role in macrophage regulation. Numerous studies have confirmed that macrophage polarization was involved in the progression and malignancy of HCC [18]. To clarify whether ACCS can impact the polarization of macrophages, we used flow cytometry to identity the composition of macrophages in tumor cells. The results showed that in tumor tissue, the phenotype of macrophages was mainly M2, and only a small proportion of macrophages was M1 (Figure 3(a)). CTS showed the ability in elevating the proportion of M1 (Figure 3(b)) while ATO gives a trend in elevating both M1 and M2 macrophages (Figures 3(b)–3(c)). More important, ACCS could greatly improve the polarization of M1 macrophages which can dramatically transfer the proportion of M1/M2 (Figure 3(d)). These results provide strong experimental evidence that ACCS can regulate tumor immunity by remodeling TAMs. To further demonstrate our results, we undertook an in vitro model of macrophages to reproduce this phenomenon. Primary macrophages were extracted from peritoneal cavities of mice, and drugs were used to stimulate macrophages for 48 h to observe the cell morphology and function. The image was captured by microscopy, and the result showed that both ATO and CTS can be stimulators to mature the primary macrophages (Figure 3(e)). Further analysis indicates that both drugs can activate the M1 polarization of macrophages, especially CTS which showed better activation to M1 and repression ability to M2. Consistent with the results in vivo, ACCS gave a more efficient activating ability to activate the M1 polarization of macrophages (Figures 4(a)–4(c)). Further attempts were conducted to prove whether ACCS can function in tumor-like macrophages. We used 10 ng/mL IL-4 to establish a tumor-like status with a high expression of M2 macrophages. Half of macrophages were forced to the M2 phenotype after modeling, and ACCS greatly inhibits the transformation of macrophages induced by IL-4 (Figures 4(d)–4(f)). In addition, ACCS gave a more efficient activating ability to transfer the ration of M1/M2 in the two groups (Figures 4(g)–4(h)). All the results indicate that ATO can accelerate the activation of macrophages, CTS specifically activates M1 and suppresses M2, and the ACCS combination can remarkably promote the polarization of M1 and elevate the ration of M1/M2, thus creating a favorable immune microenvironment for HCC treatment.

### 3.4. ACCS Regulates Glucose Metabolism in Macrophages and Tumor Tissue

**Figure 5:**
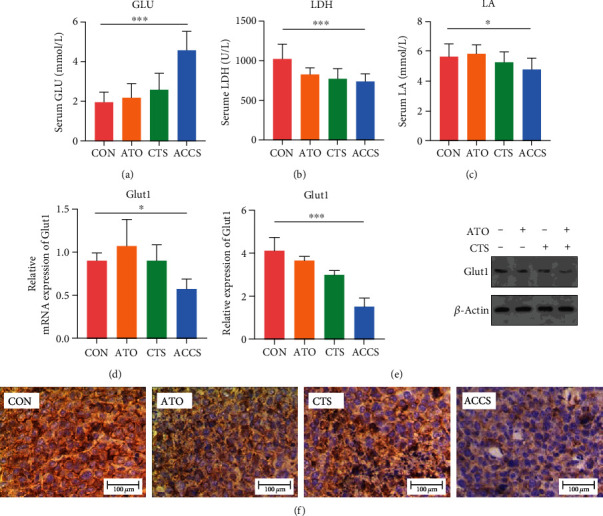
The effect of ACCS on glycolysis in HCC mice. (a) Serum glucose level. (b) Serum lactate dehydrogenase level. (c) Serum lactic acid level. (d, e) The mRNA level and the protein level of Glut1 in tumor tissue. (f) Immunohistochemical staining of Glut1 in tumor tissue.

We want to further understand the mechanisms as to why ACCS can regulate the polarization of macrophages and the reason for the superiority in combination. In the blood biochemical examination of mice, we found a significant increase in serum glucose levels after ACCS treatment (Figure 5(a)). It is well known that the Warburg effect is the feature of metabolic dysregulation in malignancy, leading to a large consumption of glucose and the occurrence of anaerobic glycolysis. Lactic acid (LA), as the main intermediate of glycolysis, can symbolize the strength and enhancement of glycolysis. Lactate dehydrogenase (LDH), as one of the enzyme systems of anaerobic glycolysis, can catalyze the catabolysis of lactate. Accordingly, we examined the levels of LDH and LA to assess glycolysis in mice. The results showed that LA and LDH were significantly reduced after ACCS treatment (Figures 5(b)–5(c)), suggesting that ACCS decreased glycolysis and lactate secretion in tumor tissues. Further, we examined the expression of Glut1, which is a key gene responsible for glucose uptake in glycolysis. The results showed that ACCS reduced the gene and protein levels of Glut1 in HCC (Figures 5(d)–5(f)). Together, these findings suggested that ACCS decreases glucose uptake and glycolysis in HCC mice.

**Figure 6:**
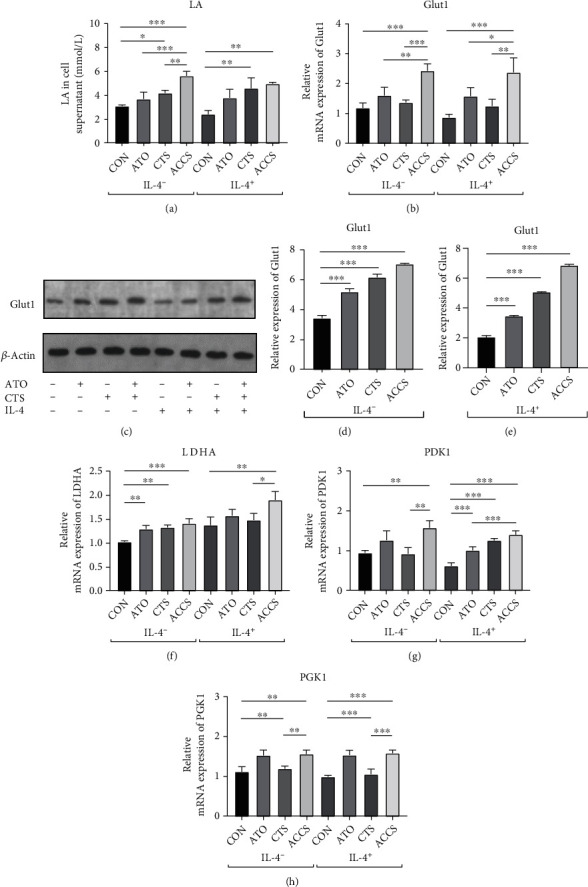
Regulation of glycolysis in primary macrophages by ACCS intervention. (a) Levels of LA in the cell supernatant. (b) Levels of Glut1 gene expression. (c–e) Levels of Glut1 protein expression. (f–h) Gene expression levels of key rate-limiting enzymes in glycolytic metabolism.

Equal to tumor cells, macrophages are also thought to be closely involved in glycolysis. Glucose metabolism was considered to be a distinct feature for different types of macrophages. To detect the potential disorders in glucose metabolism resulting from ACCS, we first detected the LA in the supernatant of macrophages, and the results showed that the level of LA was enhanced in the ACCS group (Figure 6(a)). This testifies that ACCS can increase the level of glycolysis in macrophages. To validate this hypothesis, we detected the expression of Glut1, a key gene involved in glycolysis. The results showed that ACCS can obviously increase the mRNA expression of Glut1 in two types of macrophages, and the effects are more significant than using separately (Figures 6(b)–6(e)). The protein level also confirmed a significant elevation in Glut1 after drug treatment. Analogously, we detected the mRNA expression of several glycolysis-related genes including LDHA, PDK1, and PGK1. The result showed that these glycolysis-related genes increased to varying degrees after ACCS treatment (Figures 6(f)–6(h)). All these results indicated that glycolysis changes caused by ACCS were responsible for the alteration of macrophage polarization. ACCS can speed up the uptake of glucose through Glut1 and increase the expression of glycolysis enzymes, thereby accelerating the glycolysis in macrophages.

### 3.5. ACCS Accelerates Glycolysis by Activating AMPK Signaling Pathway

**Figure 7:**
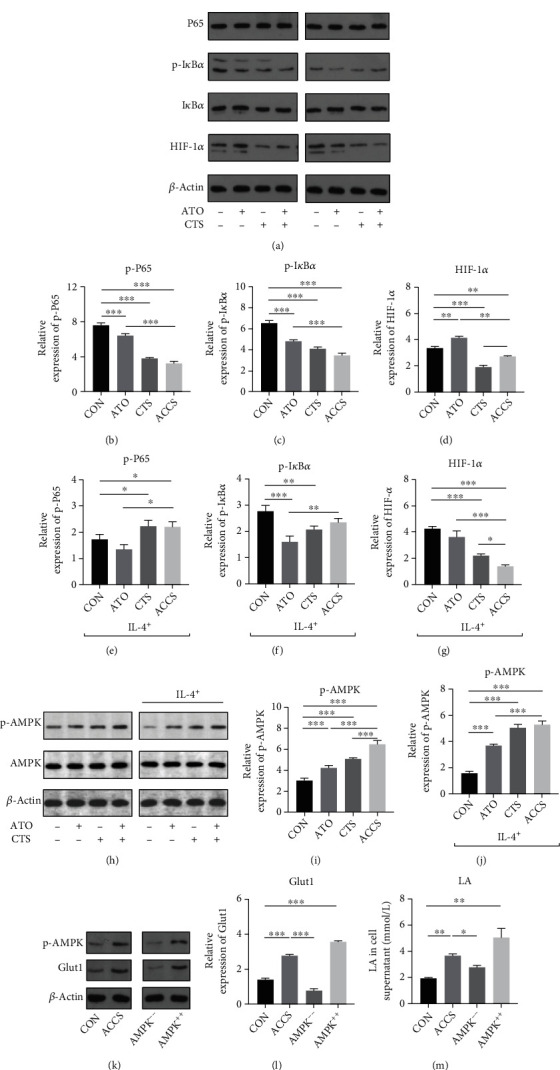
(a) Regulation of NF-*κ*B/HIF-1*α* signaling pathway by ACCS. (b–d) Activation of NF-*κ*B/HIF-1*α* signaling pathway after ACCS intervention in primary M0 macrophages. (e–g) Activation of NF-*κ*B/HIF-1*α* signaling pathway after ACCS intervention in tumor-like M2-type macrophages. (h) Regulation of AMPK signaling pathway by ACCS. (i) Activation of the AMPK signaling pathway after ACCS intervention in primary M0 macrophages. (j) Activation of AMPK signaling pathway after ACCS intervention in tumor-like M2-type macrophages. (k–m) Expression of LA in supernatant and protein level of Glut1 after AMPK blockade and activation.

Prior studies have shown that glycolysis is the main metabolic way and energy supply for M1 macrophages [12]. The activation of NF-*κ*B/HIF-1*α* and the AMPK signaling pathway is considered to be responsible for the overexpression of Glut1 and accelerated glycolysis in M1 polarization [19]. To further investigate the underlying mechanism, we first detected the level of HIF-1*α* and the phosphorylation level of NF-*κ*B p65 and p-I*κ*B*α* to characterize the activation of the NF-*κ*B/HIF-1*α* signaling pathway. Different from the typical understanding, in M0 macrophage, ACCS significantly inhibits the expression level of HIF-1*α*, NF-*κ*B p65, and p-I*κ*B*α* (Figures 7(a)–7(c)). In particular, CTS showed a more powerful inhibition to the generation of the HIF-1*α* protein (Figure 7(d)). Of note, in the IL-4-induced M2 macrophage model, ACCS promotes the expression of NF-*κ*B p65 and p-I*κ*B*α* (Figures 7(e)–7(f)) and inhibits the protein level of HIF-1*α* (Figure 7(g)). This indicates that in different types of macrophages, ACCA shows different adjustments to the HIF-1*α*/NF-*κ*B pathway.

In light of the aforementioned findings, we shifted the focus on the AMPK signaling pathway which is also responsible for the transcription of Glut1. The results testified that not only in inactivated M0 macrophages but also in M2 macrophages, ACCS can significantly activate the AMPK signaling pathway by increasing the phosphorylation of the AMPK protein (Figures 7(h)–7(j)). This revealed that AMPK activation induced by ACCS may be responsible for Glut1 elevation and glycolysis in macrophages. For further validation, we used AMPK inhibitors and activator to prove these results. The outcomes were consistent with our supposition; the inhibition of AMPK extremely decreased the protein level of Glut1 and LA in the supernatant. Accordingly, with the addition of AMPK activators, the protein level of Glut1 and LA enhanced significantly (Figures 7(k)–7(m)). All of the preceding studies indicate that the activation of AMPK, but not NF-*κ*B/HIF-1*α*, is the key point for ACCS to elevate the uptake of glucose and performing glycolysis in macrophages, thus supporting the polarization of macrophages to the M1 phenotype.

## 4. Discussion

ATO is the first-line drug for leukemia and is gaining recognition as a potential drug in solid tumors. In APL, combined ATRA-ATO therapies have manifested great success [20, 21]. From that viewpoint, hunting for the correct combination of drugs is a realizable shortcut for using ATO in tumors more broadly. Herbal medication, one of the alternative management options in clinics, is one of the resources worthy to be explored for new treatment in tumors.

*Salvia miltiorrhiza*, an herb widely used in China, is frequently used to treat cardiovascular diseases because of the function in enhancing blood circulation. The compound Danshen Dripping Pills, which is a commercialized product, is the effective ingredients extracted from *Salvia miltiorrhiza*, and it is widely applicable in the clinical setting. As the study progressed in recent years, a large amount of evidence shows that *Salvia miltiorrhiza* extracts have anti-inflammatory and antitumor activities. Among these extracts, CTS is used in HCC treatment for many researches, and our previous studies also confirmed its effectiveness [6]. We defined the combination of CTS and ATO as ACCS and focus its potential power in HCC treatment. We found that ACCS could inhibit the progression of HCC effectively both in vitro and in vivo. Our research found that the use of CTS with optimal rational dosage can significantly minimize the usage of ATO. This is very valuable for solid tumor treatment as the application of ATO has been greatly limited due to its toxicity. Our discovery broadens the use of ATO so that it can still be used in certain special cases. Not only that, but our results also partly indicate that CTS is effective in reducing the side effects of ATO, which also facilitates the further in-depth application of ATO in solid tumors.

Immediately afterwards, we went on to explore the mechanism of synergy between ATO and CTS. Based on the cues from blood testing, we identified macrophages as a potential therapeutic target for ACCS treatment. It is widely accepted that TAMs are a major component of the tumor microenvironment (TME) and play a critical role in the progression of HCC [22]. In response to inflammation or other stimulations, monocytes are recruited substantially around the injured tissue and differentiate into macrophages with different states of polarization activation, namely, M1 and M2 macrophages [23]. The M1 phenotype is a classically activated macrophage with proinflammatory properties and a high antigen-presenting capacity and promotes T cell function [24]. The M2 phenotype is an alternatively activated macrophage with excellent anti-inflammatory activity and immunomodulatory functions and can promote tissue repair in response to injury [25]. Unfortunately, macrophages frequently undergo an M2 to M1 phenotype transition during progression in HCC [26, 27]. M2 macrophages promote cancer cell proliferation and invasion through biological activities such as promoting angiogenesis and suppressing the adaptive immune system [18]. The modulation of immune cells has been a hot topic in the field of oncology therapy. The advent of PD-1 inhibitors has successfully brought T cells back from espionage to tumor killers. Our data suggested that ACCS shows the ability to increase the polarization of M1 macrophages and decrease the polarization of M2 macrophages, which can significantly reverse the ratio of M1/M2. The ability of ACCS to modulate macrophages provides additional drug options for tumor immune remodeling. In addition, related studies have clarified that ACCS is also involved in the regulation of T cells [28–30], which also provides a new strategy for tumor drug administration, such as the use of drug combinations for the coregulation of multiple immune cells.

Further elucidation of the action of ACCS will help us to better study the drug for subsequent in-depth studies. We explored its mechanism and found that glucose metabolic reprogramming is one of the main mechanisms by which it acts. It is known that the main metabolic features of M1 macrophages are increased glucose uptake and increased glycolysis, whereas M2 makes a feature of OXPHOS [12]. In this process, Glut1 plays a pivotal role. Glut1 is a member of the glucose transporter protein family that regulates the glucose transport across the cell membrane [31]. Homoplastically, the expression of glycolysis-related kinases also regulated glycolysis such as glycolytic pyruvate PDK1, LDHA, and PGK1 [32, 33]. In the IL-4-induced M2 model, we observed a decrease in Glut1 and the related kinase PDK1, LDHA, and PGK1. This implies a diminished glycolytic capacity and a shift in the metabolic phenotype. The ability of ACCS to regulate macrophage metabolism would be valuable for future investigation, as metabolic abnormalities are common in TME. Tumor cells have the ability to plunder nutrients from other cells or tissues and thus enhance the tumor nutrient supply. The predatory nature of tumors forces adaptive metabolic changes in surrounding immune cells, and metabolic reprogramming regulated by ACCS has a remodeling effect on glucose redistribution in TME.

Glycolysis is regulated by several signal transduction pathways. Among them, NF-*κ*B/HIF-1*α* and AMPK signaling pathways are two key signaling pathways that lead to changes in glycolysis [34–36]. In hepatocellular carcinoma cells, NF-*κ*B and HIF-1*α* pathways are usually in an activated state, especially under hypoxic conditions [37]. Hypoxia leads to overexpression of HIF-1*α* protein, overexpression, and nuclear translocation of NF-*κ*B, resulting in transcription of downstream genes including Glut1. Thus, enhanced glycolysis in hepatocellular carcinoma cells is largely dependent on NF-*κ*B/HIF-1*α* activation caused by hypoxia. In TAMs, the regulation of glycolysis has not been fully elucidated. We first speculated that enhanced glycolysis in macrophages was also associated with NF-*κ*B/HIF-1*α* activation, and then, unexpectedly, ACCS had a significant inhibitory effect on the NF-*κ*B/HIF-1*α* pathway in macrophages, especially in M2 macrophages. Based on this result, we speculate that the AMPK signaling pathway may be another important pathway regulated by ACCS. AMPK is one of the core molecules regulating biological energy metabolism and is capable of sensing cellular metabolic status. At low nutrient and energy levels, AMPK is activated to suppress anabolism and promote catabolism, thus maintaining intracellular energy homeostasis. At the same time, AMPK activation also regulates the glycolytic process by participating in glucose transport and inhibiting glycogen synthesis and gluconeogenesis [38]. Our study ascertained that ACCS exerts a strong activating effect on AMPK in both M0 and M2 macrophage models. Complementary studies also demonstrate that the blockage of p-AMPK can significantly weaken glycolysis. This result is interesting, as we originally thought that ACCS regulates glycolysis in tumor cells and macrophages with the same molecular mechanism, but the cellular complexity is not always as expected. This result is more helpful to elucidate why ACCS can better suppress hepatocellular carcinoma. In the microenvironment in which tumor cells are located, the enhanced glycolytic uptake by tumor cells is highly dependent on the NF-*κ*B/HIF-1*α* hypoxic pathway. As a consequence, the inhibition of the HIF-1*α* pathway by ACCS is beneficial for tumor treatment. Meanwhile, due to the activation function of ACCS on AMPK, HIF-1*α*-independent glycolysis in macrophages is consequently enhanced. This would boost energy uptake in macrophages and promote M1 type polarization, which facilitates a new balance of glucose uptake and glucose metabolism between tumor cells and immune cells.

In conclusion, abnormal energy metabolism exists not only in tumor cells but also in the immune cells around the tumor. Here, we find that the antitumor TAMs are fired in TME because they cannot absorb energy in the original way of energy harvesting. The transformed M2 macrophages sacrificed their glucose uptake ability to leave more glucose to the tumor. The use of ACCS allows macrophages to regain glucose through the AMPK signaling pathway to maintain the normal glycolysis process. This is very valuable for regulating the tumor and its surrounding cells. Previous studies often focused on the tumor itself. Now, we need to discover more about the changes in the surrounding environment caused by the tumor. This will have important supplementary value for studying the panoramic changes in tumor progression. Our research has discovered the antitumor combination of ACCS. They can regulate tumor-associated macrophages by regulating the metabolism of the tumor microenvironment to coordinate antitumor effects. We also have to admit that there are still many ambiguities in the energy competition between tumor cells and macrophages. Our research still has limitations in clarifying the relationship between the two, and our follow-up research will continue to reveal the nature of these phenomena.

## 5. Conclusions

Arsenic trioxide cooperate cryptotanshinone (ACCS) is a promising drug combination therapy in the treatment of HCC. ACCS could inhibit HCC effectively in vitro and in vivo. The therapeutic mechanism involves regulating macrophage polarization, adjusting glucose metabolism, and accelerating glycolysis in macrophages by activating the AMPK signaling pathway.

## Figures and Tables

**Table 1:** The primer of genes.

Gene name	Primer sequences(5′ to 3′)	Size (bp)
LDHA	CCTGTGTGGAGTGGTGTGA CACTGTCCACCACCTGCTT	118
PDK1	GCGACAAGAGTTGCCTGTTAGATT GGATGGGGTCCTGAGAAGA	82
PGK1	CGACCCTTCCTGGCTATCTT CAATCTCCATGTTGTTGAGCACCTT	145
GLUT1	GTGTATCCTGTTGCCCTTCTG CTGCCGACCCTCTTCTTTC	151

**Table 2:** Blood routine examination of mice.

Description	NC (*n* = 8)	ATO (*n* = 8)	CTS (*n* = 8)	ACCS (*n* = 8)
WBC (10^9^/L)	6.34 ± 1.36	7.83 ± 2.39	7 ± 1.58	8.99 ± 3.47^∗^
RBC (10^12^/L)	10.85 ± 0.63	9.86 ± 1.01^∗^	10.6 ± 1.1	9.64 ± 1.19^∗^
HGB (g/L)	153.38 ± 7.76	143.88 ± 13.09	151.5 ± 14.5	143.63 ± 17.76
HCT (%)	50.05 ± 1.95	47.2 ± 3.6	49.15 ± 4.57	46.3 ± 4.8^∗^
MCV (fL)	46.2 ± 1.35	47.99 ± 1.47^∗^	46.41 ± 0.86	48.33 ± 1.1^∗∗^
MCH (pg)	14.15 ± 0.21	14.61 ± 0.33^∗∗^	14.31 ± 0.2	14.76 ± 0.13^∗∗∗^
MCHC (g/L)	306.38 ± 4.93	304.63 ± 5.32	308.25 ± 3.85	306.63 ± 7.27
PLT (10^9^/L)	2127 ± 375.87	2249 ± 276.96	1831.88 ± 51.43^∗^	2476.25 ± 392.99^∗^
RDW-SD (fL)	26.16 ± 2.36	29.15 ± 1.34^∗∗^	24.18 ± 1.54^∗^	27.78 ± 1.12
RDW-CV (%)	19.08 ± 0.95	19.11 ± 0.87	18.05 ± 0.56^∗^	18.21 ± 0.7^∗^^△^
PDW (fL)	6.51 ± 0.17	6.53 ± 0.13	6.46 ± 0.11	6.59 ± 0.12
MPV (fL)	6.3 ± 0.19	6.39 ± 0.21	6.29 ± 0.2	6.44 ± 0.29
P-LCR (%)	2.71 ± 0.97	3.18 ± 0.69	2.81 ± 0.76	3.83 ± 1.52
PCT (%)	1.28 ± 0.33	1.44 ± 0.21	1.15 ± 0.04	1.64 ± 0.32^∗^
NRBC# (10^9^/L)	0.02 ± 0	0.01 ± 0.01	0.03 ± 0.02	0.02 ± 0.01
NRBC% (%)	0.29 ± 0.08	0.15 ± 0.14^∗^	0.35 ± 0.17	0.31 ± 0.28
NEUT# (10^9^/L)	1.09 ± 0.78	2.14 ± 1.73	1.65 ± 1.35	3.49 ± 2.47^∗^
LYMPH# (10^9^/L)	4.92 ± 1.23	5.2 ± 0.88	5.1 ± 0.95	5.83 ± 2.19
MONO# (10^9^/L)	0.22 ± 0.16	0.32 ± 0.31	0.18 ± 0.16	0.54 ± 0.36^∗^
EO# (10^9^/L)	0.11 ± 0.06	0.17 ± 0.14	0.07 ± 0.06	0.18 ± 0.13
BASO# (10^9^/L)	0.01 ± 0.01	0.01 ± 0.01	0 ± 0.01	0.01 ± 0.01
NEUT% (%)	16.78 ± 9.83	24.23 ± 14.03	21.88 ± 12.42	31.94 ± 20^∗^
LYMPH% (%)	78.26 ± 11.84	70.25 ± 17.18	74.81 ± 14.19	62.25 ± 24.02
MONO% (%)	3.3 ± 1.96	3.5 ± 2.48	2.39 ± 1.46	4.7 ± 3.23
EO% (%)	1.6 ± 0.82	1.96 ± 1.13	0.86 ± 0.61^∗^	1.59 ± 1.15
BASO% (%)	0.06 ± 0.09	0.06 ± 0.07	0.06 ± 0.09	0.08 ± 0.08
NLR	0.24 ± 0.23	0.42 ± 0.35	0.35 ± 0.34	0.77 ± 0.84
PLR	401.3 ± 225.57	447.23 ± 106.35	370.01 ± 67.84	526.01 ± 281.97
LMR	30.17 ± 13.38	34.23 ± 25.03	42.2 ± 21.82	24.63 ± 24.83

^∗^Compared to the NC group, ^∗^<0.05, ^∗∗^<0.01, ^∗∗∗^<0.001; compared to the ATO group, ^△^<0.05.

## Data Availability

The data used to support the findings of this study are available from the corresponding authors upon request.

## References

[1] Global Burden of Disease Liver Cancer Collaboration, Akinyemiju T., Abera S. (2017). The burden of primary liver cancer and underlying etiologies from 1990 to 2015 at the global, regional, and national level: results from the Global Burden of Disease Study 2015. *JAMA Oncology*.

[2] Xiaoqin C., Zhang R., Zhao T. (2019). Targeted arsenite-loaded magnetic multifunctional nanoparticles for treatment of hepatocellular carcinoma. *Nanotechnology*.

[3] Teran V. A., Wilson B. B., Guffey D. J. (2019). Flexural eruption associated with arsenic trioxide therapy in a patient with acute promyelocytic leukemia. *JAMA Dermatology*.

[4] Akhtar A., Wang S. X., Ghali L., Bell C., Wen X. (2017). Recent advances in arsenic trioxide encapsulated nanoparticles as drug delivery agents to solid cancers. *Journal of Biomedical Research*.

[5] Fei W., Zhang Y., Han S. (2017). RGD conjugated liposome-hollow silica hybrid nanovehicles for targeted and controlled delivery of arsenic trioxide against hepatic carcinoma. *International Journal of Pharmaceutics*.

[6] Li S., Zhang G., Lou Z., Xu G., Zhang G. (2016). *Cryptotanshinone sensitizes arsenic trioxide-induced Bel-7404 liver cancer cell apoptosis by downregulating phosphorylated-STAT3 in vitro and in vivo*.

[7] Mann J. (2002). Natural products in cancer chemotherapy: past, present and future. *Nature Reviews. Cancer*.

[8] Wang K., Jin X., Li Q. (2018). Propolis from different geographic origins decreases intestinal inflammation and Bacteroides spp. populations in a model of DSS-induced colitis. *Molecular Nutrition & Food Research*.

[9] Wang K., Wan Z., Ou A. (2019). Monofloral honey from a medical plant, Prunella vulgaris, protected against dextran sulfate sodium-induced ulcerative colitis via modulating gut microbial populations in rats. *Food & Function*.

[10] Ni S., Liao G., Zhang G. (2015). The effects of compatibility of arsenic trioxide and cryptotanshinone on human hepatocarcinoma nude mice. *Journal of Zhejiang Chinese Medical University*.

[11] Zhang Y. L., Li Q., Yang X. M. (2018). SPON2 promotes M1-like macrophage recruitment and inhibits hepatocellular carcinoma metastasis by distinct integrin-rho GTPase-Hippo pathways. *Cancer Research*.

[12] Viola A., Munari F., Sánchez-Rodríguez R., Scolaro T., Castegna A. (2019). The metabolic signature of macrophage responses. *Frontiers in Immunology*.

[13] Han Z., Liu S., Lin H., Trivett A. L., Oppenheim J. J. (2019). Inhibition of murine hepatoma tumor growth by cryptotanshinone involves TLR7-dependent activation of macrophages and induction of adaptive antitumor immune defenses. *Cancer Immunology, Immunotherapy*.

[14] Yang Y., Cao Y., Chen L. (2018). Cryptotanshinone suppresses cell proliferation and glucose metabolism via STAT3/SIRT3 signaling pathway in ovarian cancer cells. *Cancer Med*.

[15] Sun R. C., Board P. G., Blackburn A. C. (2011). Targeting metabolism with arsenic trioxide and dichloroacetate in breast cancer cells. *Molecular Cancer*.

[16] Subbarayan P. R., Ardalan B. (2014). In the war against solid tumors arsenic trioxide need partners. *Journal of Gastrointestinal Cancer*.

[17] Chou T. (2010). Drug combination studies and their synergy quantification using the Chou-Talalay method. *Cancer Research*.

[18] Tian Z., Hou X., Liu W., Han Z., Wei L. (2019). Macrophages and hepatocellular carcinoma. *Cell & Bioscience*.

[19] D’Ignazio L., Bandarra D., Rocha S. (2016). NF-*κ*B and HIF crosstalk in immune responses. *FEBS Journal*.

[20] Gore S. D., Gojo I., Sekeres M. A. (2010). Single cycle of arsenic trioxide-based consolidation chemotherapy spares anthracycline exposure in the primary management of acute promyelocytic leukemia.. *Journal of Clinical Oncology*.

[21] Kutny M. A., Alonzo T. A., Gerbing R. B. (2017). Arsenic trioxide consolidation allows anthracycline dose reduction for pediatric patients with acute promyelocytic leukemia: report from the Children’s Oncology Group Phase III Historically Controlled Trial AAML0631. *Clinical Oncology*.

[22] Ostuni R., Kratochvill F., Murray P. J., Natoli G. (2015). Macrophages and cancer: from mechanisms to therapeutic implications. *Trends in Immunology*.

[23] Sica A., Mantovani A. (2012). Macrophage plasticity and polarization: in vivo veritas. *The Journal of Clinical Investigation*.

[24] Galli S. J., Borregaard N., Wynn T. A. (2011). Phenotypic and functional plasticity of cells of innate immunity: macrophages, mast cells and neutrophils. *Nature Immunology*.

[25] Sica A., Invernizzi P., Mantovani A. (2014). Macrophage plasticity and polarization in liver homeostasis and pathology. *Hepatology*.

[26] Schreiber R. D., Old L. J., Smyth M. J. (2011). Cancer immunoediting: integrating immunity’s roles in cancer suppression and promotion. *Science*.

[27] Prieto J., Melero I., Sangro B. (2015). Immunological landscape and immunotherapy of hepatocellular carcinoma. *Nature Reviews. Gastroenterology & Hepatology*.

[28] Wang L., Hu X., Xu Y., Liu Z. (2016). Arsenic trioxide inhibits lung metastasis of mouse colon cancer via reducing the infiltration of regulatory T cells. *Tumor Biology*.

[29] Haque R., Chaudhary A., Sadaf N. (2017). Immunomodulatory role of arsenic in regulatory T cells. *Endocrine, Metabolic & Immune Disorders - Drug Target**s***.

[30] Man Y., Yang L., Zhang D., Bi Y. (2016). Cryptotanshinone inhibits lung tumor growth by increasing CD4+ T cell cytotoxicity through activation of the JAK2/STAT4 pathway. *Oncology Letters*.

[31] Deng D., Xu C., Sun P. (2014). Crystal structure of the human glucose transporter GLUT1. *Nature*.

[32] Adeva M., González-Lucán M., Seco M., Donapetry C. (2013). Enzymes involved in l-lactate metabolism in humans. *Mitochondrion*.

[33] Menard L., Maughan D., Vigoreaux J. (2014). The structural and functional coordination of glycolytic enzymes in muscle: evidence of a metabolon?. *Biology (Basel).*.

[34] Li J., Zhang J., Xie F., Peng J., Wu X. (2018). Macrophage migration inhibitory factor promotes Warburg effect via activation of the NF-*κ*B/HIF-1*α* pathway in lung cancer. *International Journal of Molecular Medicine*.

[35] D’Ignazio L., Batie M., Rocha S. (2017). Hypoxia and inflammation in cancer, focus on HIF and NF-*κ*B. *Biomedicine*.

[36] Faubert B., Boily G., Izreig S. (2013). AMPK is a negative regulator of the Warburg effect and suppresses tumor growth in vivo. *Cell Metabolism*.

[37] Feng W., Xue T., Huang S. (2018). HIF-1*α* promotes the migration and invasion of hepatocellular carcinoma cells via the IL-8–NF-*κ*B axis. *Cellular & Molecular Biology Letters*.

[38] Kelly B., O'Neill L. A. J. (2015). Metabolic reprogramming in macrophages and dendritic cells in innate immunity. *Cell Research*.

